# Comparing saliva and blood for the detection of mosaic genomic abnormalities that cause syndromic intellectual disability

**DOI:** 10.1038/s41431-022-01232-5

**Published:** 2022-11-29

**Authors:** David I. Francis, Zornitza Stark, Ingrid E. Scheffer, Tiong Yang Tan, Krithika Murali, Lyndon Gallacher, David J. Amor, Himanshu Goel, Lilian Downie, Chloe A. Stutterd, Emma I. Krzesinski, Anand Vasudevan, Ralph Oertel, Vida Petrovic, Amber Boys, Vivian Wei, Trent Burgess, Karen Dun, Karen L. Oliver, Anne Baxter, Anna Hackett, Samantha Ayres, Sebastian Lunke, Paul Kalitsis, Meaghan Wall

**Affiliations:** 1 Victorian Clinical Genetics Services, Murdoch Children’s Research Institute, Flemington Road, Melbourne, VIC Australia; 2 University of Melbourne, Melbourne, VIC Australia; 3 Austin Health and Royal Children’s Hospital, Heidelberg and Parkville, VIC Australia; 4 Florey Institute of Neuroscience and Mental Health, Parkville, VIC Australia; 5 Murdoch Children’s Research Institute, Flemington Road, Melbourne, VIC Australia; 6 Hunter Genetics, Waratah, NSW Australia; 7 University of Newcastle, Callaghan, NSW Australia; 8 Monash Health, Monash Medical Centre, Clayton, VIC Australia; 9 Royal Women’s Hospital, Parkville, VIC Australia; 10 Victorian Cancer Cytogenetic Service, St Vincent’s Hospital, Victoria Parade, Melbourne, VIC Australia

**Keywords:** Medical genomics, Cytogenetics

## Abstract

We aimed to determine whether SNP-microarray genomic testing of saliva had a greater diagnostic yield than blood for pathogenic copy number variants (CNVs). We selected patients who underwent CMA testing of both blood and saliva from 23,289 blood and 21,857 saliva samples. Our cohort comprised 370 individuals who had testing of both, 224 with syndromic intellectual disability (ID) and 146 with isolated ID. Mosaic pathogenic CNVs or aneuploidy were detected in saliva but not in blood in 20/370 (4.4%). All 20 individuals had syndromic ID, accounting for 9.1% of the syndromic ID sub-cohort. Pathogenic CNVs were large in size (median of 46 Mb), and terminal in nature, with median mosaicism of 27.5% (not exceeding 40%). By contrast, non-mosaic pathogenic CNVs were 100% concordant between blood and saliva, considerably smaller in size (median of 0.65 Mb), and predominantly interstitial in location. Given that salivary microarray testing has increased diagnostic utility over blood in individuals with syndromic ID, we recommend it as a first-tier testing in this group.

## Introduction

In the last 15 years, whole-genome microarray testing has superseded conventional G-banded chromosomal analysis and broadened the detection limits to include cryptic unbalanced chromosome abnormalities and copy number variants (CNVs) (>50 kb) [1]. SNP chromosomal microarray (CMA) is the investigation of choice in individuals with syndromic and non-syndromic intellectual disability (ID), neurodevelopmental disorders and multiple congenital anomalies [2]. Microarray techniques use good quality DNA from diverse tissues (blood, buccal, skin fibroblast, amniocytes and chorionic villi), allowing testing in different clinical scenarios and enabling the detection of mosaicism. Many studies have demonstrated that SNP-microarray technology detects CNV mosaicism in 0.35–1.0% of affected individuals tested [2–4].

Recently, saliva samples have been utilised for both genetic and non-genetic diagnostic applications in medicine (e.g., detection of infectious agents, metabolites, drug and alcohol levels), proving to be a cost-effective and convenient way of sampling that provides equivalent diagnostic yield to blood [5, 6]. Moreover, saliva contains a range of cell types including leucocytes, and cells of mesodermal and ectodermal origin [7]. In particular, buccal or fibroblast cells, that are present in saliva in variable amounts/quantities [8], are the best tissue for the detection of mosaicism for aneuploidy or CNVs. [9]. Genome sequencing (GS) or exome sequencing (ES) in patients with undiagnosed developmental disorder (UDD) detected somatic mosaic gains or losses more frequently in saliva (9/3246, 0.28%) than blood (2/1652, 0.12%) [10], but this approach has not been tested using SNP-microarrays.

Here, we aimed to compare the yield of SNP-microarray technology in saliva compared with blood to detect low-level aneuploidy or CNV mosaicism in patients with intellectual disability. We retrospectively interrogated SNP-microarray results in patients who had provided both a peripheral blood sample and saliva sample. We analysed the features of the aneuploidy and CNVs detected to understand which were more likely to be detected in saliva compared with blood.

## Methods

Patients were referred to the clinical diagnostic laboratory of VCGS, Melbourne, Australia. Clinical data were extracted from the VCGS database for diagnostic case reporting and storage. Clinical data were classified based on the clinical features in the laboratory referral into syndromic ID (where ID was associated with dysmorphic features or other congenital abnormalities) and non-syndromic ID. As many of the 370 patients’ ages at clinical assessment were less than 5 years old, they were not formally diagnosed with intellectual disability but had developmental delays recorded. Please note that clinical information was always obtained from clinical referrals (unstructured clinical indication field). If no clinical details were supplied the clinician was contacted for the clinical indication. Any referrals with insufficient clinical details to determine classification of syndromic ID/non-syndromic ID were excluded from this study.

Saliva samples were collected using a paediatric saliva kit (DNA Genotek, Ottawa, Ontario Canada, PN OC-175) following the manufacturer’s protocol. Salivary or blood DNA was extracted using a Qiagen Symphony automated DNA extraction protocol.

DNA was processed using the Illumina (San Diego, California) Infinium workflow on the Core Exome-24 BeadChip (650 k SNP probes) or Global Screening Array (GSA) -24v1 or v2 (650 k SNP probes) BeadChips. Both platforms had a mean probe spacing of 6 kb and were validated for the detection of deletions and duplications to 200 kb and/or 20 probes. The minimum 20 probe threshold for CNV calling allowed for detection of CNVs to 1 kb in size. Sensitivity for mosaicism was validated to detect CNVs (whole chromosome, segmental chromosome (>10 Mb) and smaller copy number variants (<10 Mb)) at the 5–10% level or greater. The percentage of mosaicism is determined as the mean variant allele fraction (VAF). Copy number analysis was performed using the Illumina Karyostudio v1.4 or BioDiscovery NxClinical v6.0. Classification of CNVs detected by CMA was based on the Human Genetics Society of Australasia (HGSA) “Best Practice Guidelines for Chromosomal Microarray for Australasian Laboratories” (https://www.hgsa.org.au/hgsanews/microarray-best-practice-guidelines-updated) and evidence-based curation and reporting using peer reviewed publications. Genomic coordinates are provided against human genome reference GRCh37/hg19.

We analysed the type of CNV (gain or loss), size of genomic imbalance, chromosomal position of the abnormality, mosaicism level and reason for referral (syndromic ID or non-syndromic ID).

## Results

**Fig. 1:**
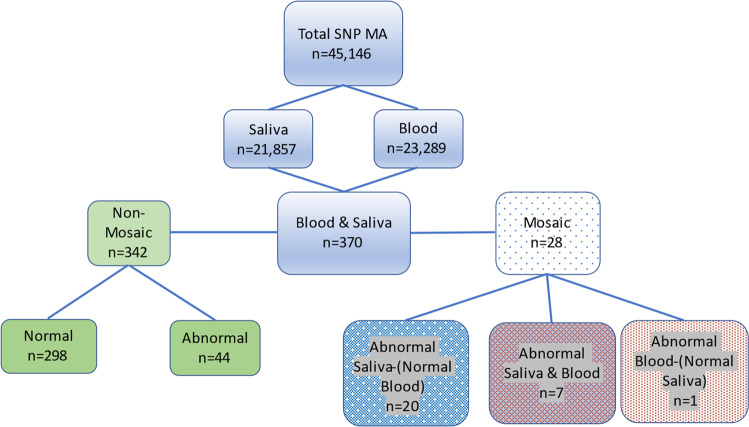
Flow diagram pipeline for SNP-microarray test results, comparing outcomes for DNA derived from saliva compared with blood. The data from all cases was separated into different sample types, cases where both sample types were tested and the different non-mosaic and mosaic classes. The mosaic class was further separated into three specific tissue mosaicism subclasses.

**Fig. 2:**
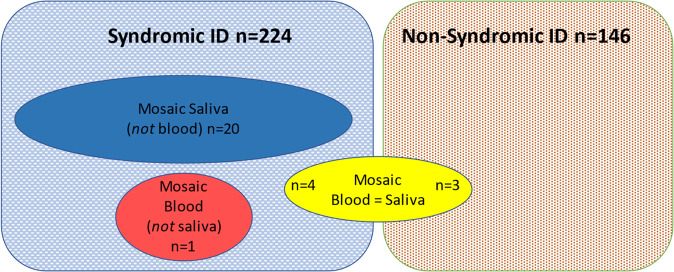
Patient cohorts with mosaic CNVs detected on SNP-microarray in saliva and/or blood. The boxes divide the 370 patients into two clinical classes; syndromic ID and non-syndromic ID. Within these clinical classes the three tissue mosaic subclasses are positioned, noting that all 20 saliva mosaic (but blood normal) cases were positioned within the syndromic ID clinical subclass.

We had SNP-microarray testing results on 23,289 blood samples and 20,857 saliva samples from 6,653 individuals with syndromic ID and 37,493 with non-syndromic ID. In 370 individuals, microarray testing had been performed in both saliva and blood (Fig. 1). This included 224 patients with syndromic ID and 146 with non-syndromic ID (Fig. 2). Where the microarray was abnormal, a second tissue was obtained to analyse tissue-specific mosaicism. There were five different scenarios of abnormal aneuploidy or CNVs detected in different tissues (Table 1 and Supplementary Table 1). There was very little difference between saliva and blood DNA quality metrics and SNP MA quality metrics. Any small differences detected had no effect on CNV calling sensitivity or specificity. Failure rates were slightly higher for saliva samples than blood sampling although in the paediatric setting this was minimal (personal communication: Dr Paul Kalitsis, publication in submission).

**Table 1:** Comparison of abnormal mosaic and non-mosaic SNP-CMA results in blood and saliva.

	Normal saliva	Mosaic saliva	Non-mosaic saliva
Normal blood	298	20 (66% gain/26% deletion) (95% terminal gain or deletion) (Gain 38 Mb/deletion 54 Mb) (mosaicism 20% for gains, 30% for losses, 10% trisomy) Syndromic ID	0
Mosaic blood	1 (trisomy 18, Syndromic ID)	7 (see Supplementary Table 2)	0
Non-mosaic blood	0	0	44 (46% gain/54% deletion) (one individual trisomy 18, syndromic ID) (83% interstitial/17% terminal) (gain 0.6 Mb/deletion 0.7 Mb)

For non-mosaic abnormal and normal microarray results, blood and saliva showed the same result. Non-mosaic genomic abnormalities comprised segmental gains (46%) and deletions (54%), with a similar median size of 0.7 Mb for deletions (range 0.05–18 Mb) and 0.6 Mb for duplications (range 0.06–41 Mb), and 83% were interstitial in location (Table 1 and Supplementary Table 1). In addition to the CNV changes, there was one case of trisomy 18.

In 20 individuals, a mosaic abnormality was observed in saliva but was not present in blood (Table 1 and Supplementary Table 1). All CNVs detected were reported as pathogenic based on gene content, genomic size and similar abnormal database entries (Decipher), although clinical relevance would be dependent on tissue distribution. This finding was only observed in the cohort with syndromic ID and not seen in those with non-syndromic ID (Fig. 2). Mosaic pathogenic variants in saliva comprised either a CNV or trisomy. Molecular abnormalities were mainly of large size (>8 Mb, except for individual 1 with partial deletion of *NIPBL*), either being trisomies 6/19 (32%) or large(>1 Mb) CNVs 13/19(67%). Of the 13 patients with large CNVs, 12/13 (92%) showed an abnormality that involved a gain, with a median size of 38 Mb (range 3–64 Mb), whilst 4/13 (30%) showed deletions that had a larger median size of 54 Mb (range 1.3–85 Mb). All pathogenic large CNV abnormalities were terminal in nature.

Overall the median VAF for all CNVs was determined at 27.5% in the 20 individuals. Specifically, patients had a median of 25% mosaicism level (range 5–40%) for both deletions and duplications. In contrast, six patients with trisomy had a median of 10% mosaicism. An additional buccal, fibroblast or organ sample was available in 7/20 cases. Interestingly, a higher percentage mosaicism was found in all tissues tested compared with saliva (Supplementary Table 1). The higher percentage of mosaicism found in all tissues compared to the saliva DNA is probably because a large percentage of saliva DNA is from haematopoietic origin, which was proven normal (blood sampling). Comparison of saliva and blood was important in determining pathogenicity in some cases. For example, case 3 had a larger ‘likely pathogenic’ mosaic (20%) 4 Mb deletion, 4q35.2(187113041_191154276)×1 and a mosaic pathogenic 85 Mb duplication 1q23.3q44(163494784–249222527)×3 in saliva, whereas, in blood only had a 1.3 Mb 40% mosaic deletion of 4q35.2(189666512–190937862)×1 that was reported as a VUS because it was small and without genes that were pathogenic for haploinsufficiency by ClinGen [11]. In case 11, there was a larger mosaic pathogenic 13 Mb deletion (10q26.11q26.3) only observed in saliva. Two duplications were seen in both blood and saliva at 2q33.1 and 11p15.1p14.3, both of which were considered to be VUS.

Conversely, there was one patient in whom a mosaic abnormality, trisomy 18, was present in blood but not saliva (Fig. 1 and Table 1). This patient was 10% mosaic for trisomy 18 and had cognitive impairment, cardiac abnormalities, and microcephaly.

For mosaic CNVs, seven individuals had the same result in saliva and blood, including an extra structurally abnormal chromosome (ESAC) and copy-neutral changes (Supplementary Table 2).

## Discussion

In this large series of individuals with syndromic intellectual disability, we show that the diagnostic yield for mosaic pathogenic CNVs is higher in saliva than blood. We found clinically relevant mosaicism for genomic changes in 9.1% (20/224) of saliva samples compared with 0.44% (1/224) in blood ((*Z* = 4.2469) *p* < 0.00001 at 5% significance level). Conversely, there was no difference in the detection of non-mosaic copy number changes between blood and saliva samples.

Mosaicism level detected in saliva, but not blood, ranged from 5 to 40% (median 27.5%) for CNVs (including trisomies). Trisomies had a lower median level of 10% mosaicism compared with a median of 25% for deletions and duplications. This lower percentage mosaicism for trisomies is consistent with their lethal outcome in the non-mosaic state, such as for trisomies 1, 7, 12, 15 and 16 [12]. Trisomy 1 is novel, and has rarely been reported postnatally or prenatally.

All large mosaic CNV abnormalities in saliva were terminal in nature, suggesting a mitotic recombination single break crossover event (terminal structural arrangements only have one genomic break compared to interstitial with two breaks). This contrasts with mosaicism in blood where CNVs are largely interstitial, with deletions being more common.

SNP-CMA technology enables detection of lower levels of mosaicism and trisomies compared with NGS approaches, genome sequencing (GS) or exome sequencing (ES) [10]. Our study, compared to King et al., detected larger median sizes for both copy number gains (38 Mb) and losses (54 Mb) compared with a previous study of UDD that detected gains (26 Mb) and losses (20 Mb) using GS/ES. We detected mosaicism at lower levels (median gain 20% and loss 30%) compared with GS/ES (median gain 55% and loss 46%), most likely reflecting that any low-level mosaicism (<50%) was detected by a previous clinical microarray, thereby excluding these patients for recruitment in the King et al. study. GS/ES techniques should have the same sensitivity as a SNP-microarray to detect these mosaic CNVs, although this may be dependent on increasing sequencing depths particularly of GS and incorporation of CNV detection tools in clinically validated pipelines.

We found that a relatively uncertain mosaic finding in blood may be part of a complex pathogenic finding, determined by testing non-hemopoietic cells. For example, there were two individuals (cases 3 and 10, Supplementary Table 1) that were reported as mosaic VUS in blood but saliva showed a larger, adjacent mosaic pathogenic CNV. Testing of a saliva sample in this scenario is recommended as blood testing may not detect the mosaic pathogenic cell line. The only mosaic abnormality detected in blood but not saliva was a low-level mosaic trisomy 18. This individual had intellectual disability, heart anomalies and microcephaly indicating mosaicism affected multiple organ systems including the brain.

It is hypothesised that the genomic abnormalities identified in saliva derive from buccal epithelial cells in a saliva sample. Epithelial cells are embryologically closer to neural cells as they are derived from ectoderm. This likely explains the higher yield in saliva testing in patients with ID than mesoderm-derived blood cells [13]. A buccal swab would be expected to have an even higher percentage mosaicism than saliva as it represents a more homogeneous sample of epithelial cells. Six cases had both buccal and salivary testing and the percentage mosaicism was always higher in the buccal samples. Testing of a skin fibroblast or buccal sample in individuals with syndromic ID may provide the best detection of ectodermal tissue-limited mosaicism. This is demonstrated by case 16, that showed 60% mosaicism in fibroblasts compared with 25% in saliva.

We infer that these mosaic abnormalities are pathogenic in these individuals with ID. The CNVs in non-mosaic form (as per Table 1) are considered pathogenic in significance using ACMG CNV curation guidelines, as is the absence of these CNVs in databases of normal variation. Furthermore in testing of over 6000 saliva samples of normal individuals (parental samples) there was no detection of mosaicism of CNVs. Ideally we would test brain tissue but such tissue is largely inaccessible. The size of the genomic imbalances supports pathogenicity, differentiating them from smaller (<1 Mb) tissue-limited mosaic CNVs found in both normal and clinically abnormal brains [13–15]. It would be of interest to study cardiac tissue, given that six individuals had cardiac anomalies. Individual 17 with mosaic trisomy 12 had their trisomy confirmed in mesodermally derived cardiomyocytes by FISH testing of a heart biopsy. This could be explained by an earlier somatic aberration affecting both ectodermal and mesodermal tissues.

For non-mosaic CNVs, results were consistent between blood and saliva, suggesting that one tissue sample is sufficient for diagnosis. The proportion of gain to deletion abnormalities was relatively even, and of comparable median size for duplications (0.6 Mb) and deletions (0.7 Mb). Non-mosaic deletions and duplications were predominantly interstitial (83%) which is consistent with a meiotic, non-allelic homologous recombination (NHR) mechanism, aligning with the non-mosaic nature of the CNV [12].

In seven individuals, mosaicism for pathogenic findings was consistent between blood and saliva. These involved both CNV and copy-neutral changes (whole and partial uniparental disomy (UPD) and chimerism) and did not affect the same genomic regions (data in Supplementary Table 2). The percentage of the abnormal cells was also relatively consistent indicating that the abnormal cell line was distributed evenly in at least these tissues and likely occurred earlier in embryological development.

A limitation of our study is its retrospective nature. Larger, prospective studies where CMA are performed on both saliva and blood samples for a range of clinical indications would further inform testing pathways. In our cohort, clinical indications of mosaicism, such as pigmentary changes, may have increased our yield due to testing of additional tissue types. Patients less than 5 years old could also not be assessed for ID.

We showed a statistically significant increase in the detection of tissue-limited mosaicism for pathogenic copy number changes in individuals with syndromic ID in saliva (9.1%) compared with blood (0.44%). Conversely, there was no difference in the detection of non-mosaic copy number pathogenic changes between blood and saliva samples. We suggest that in individuals with syndromic ID, saliva or buccal genomic testing be performed as the first-tier test over blood testing. If previous blood GS/ES or CMA testing is normal, saliva or buccal CMA (or as costs decrease WG/ES) should also be considered to detect tissue-limited low-level CNV mosaicism.

## Supplementary information

Supplementary Table 1

Supplementary Table 2

Supplementary Table 3

## Data Availability

All data generated or analysed during this study are included in this published article and its Supplementary information files.
