# Supplementary material for: Comparing saliva and blood for the detection of mosaic genomic abnormalities that cause syndromic intellectual disability
Source: Eur J Hum Genet. 2022 Nov 29;31(5):521–5. doi: 10.1038/s41431-022-01232-5 (PMC10172398; doi:10.1038/s41431-022-01232-5)
Supplement: Supplementary file 1 — Supplementary Table 1 [file 41431_2022_1232_MOESM1_ESM.docx]

**Supplementary Table 1:** Saliva tissue-limited mosaicism data in 20 individuals for genomic abnormality, percentage mosaicism and clinical features.

| **Individuals** | **Genomic abnormality [hg19]** | **Type of Abnormality/Size(Mb)**  **Loss Gain Trisomy** | | | **Mosaic %(saliva)** | **Mosaic % (buccal/**  **tissue/SF)** | **Clinical features** |
| --- | --- | --- | --- | --- | --- | --- | --- |
| 1 | 5p13.2(36,791,812-36,895,270)x1 | 0.1 |  |  | 40 | Not done | 4-yo female. Cornelia De Lange syndrome (*NIPBL* gene deletion). |
| 2 | 16p12.1p11.1(26,709,540-34,981,746)x3 |  | 8 |  | 40 | Not done | 2-yo male. Global developmental delay and facial dysmorphism. |
| 3 | 1q23.3q44(163494784-249222527)x3[saliva],  4q35.2(187113041_191154276)x1 [saliva and blood] | 4 | 85 |  | 20 | 25 | 1-yo female. IUGR, dysmorphism, short-long bones, VSD/ASD, talipes, global developmental delay. |
| 4 | 7q31.31q36.3(120,714,700-159,119,220)x3 |  | 38 |  | 20 | Not done | 3-yo male.ID, ASD, respiratory problems |
| 5 | 4q31.3q35.1(152,592,191-190,777,761)x3, 18q21.2q23(48,731,131-78,014,582)x1 | 29 | 38 |  | 30 | Not done | 35-yo male. ID, ASD, epilepsy. |
| 6 | (15)x3  trisomy 15, hmz(mat) |  |  | 81 | 15 | 25 | 26-yo male. ID, epilepsy, Prader-Willi syndrome. |
| 7 | 12p13.33p11.1(68,276-34,843,869)x2~4 |  | 64 |  | 15 | Not done | 21-yo female. Pallister Killian syndrome. |
| 8 | (21)x3  Trisomy 21 |  |  | 48 | 15 | Not done | 17-yo male. ID, ASD, Short stature. |
| 9 | 5p15.33p11(38,139-46,228,333)x3 |  | 48 |  | 5 | Not done | 8yo-female hyperpigmented skin lesions, macrocephaly, mild learning difficulties |
| 10 | 5p15.33p11(38,139-46,228,333)x3 |  | 46 |  | 20 | 50 | 17-yo male. Severe ID, dysmorphism, seizures, osteoporosis. |
| 11 | 2q33.1(202838353_203206909)x3 [saliva and blood], 10q26.11q26.3(121679013-135534747)x1 [saliva only] 11p15.1p14.3(19456304_22613634)x3 [saliva and blood] | 13 | 0.4 & 3 |  | 30/50/50 | Not done | 2-yo female. Hypotonia, Mild ID, encephalopathy. |
| 12 | 13q13.2q34(34899833_115169878)x1 | 80 |  |  | 30 | Not done | 45-yo male. ID, talipes, spinal malformation, skin pigmentation |
| 13 | 12p13.33q12(1_38547969)x3 |  | 38 |  | 5 | Not done | 11-yo male Learning difficulties, vascular birth mark, multiple hypopigmented macules, 2&3 syndactyly both feet |
| 14 | 12p13.33q12(1_38547969)x3 |  | 38 |  | 25 | Not done | 10-yo female. Mild ID and dysmorphism |
| 15 | 3q25.2q29(154200833_197871052)x3 |  | 43 |  | 40 | Not done | 4-mo male. Bilateral sensorineural hearing loss, constipation, petechial rash, patent ductus arteriosus, sacral dimple, bifid uvula, respiratory problems |
| 16 | (16)x3  Trisomy 16 |  |  | 90 | 20 | 30 | 1-mo female. Severe IUGR, idiopathic ileal dilation, VSD, ASD, persistent pulmonary hypertension. |
| 17 | (12)x3  Trisomy 12 |  |  | 132 | 5 | 8 | 3-mo female. Multiple choroid plexus cysts (up to 11mm) and prominent cisterna magna, dysmorphic features and cardiac abnormalities |
| 18 | 11q23.3q25(120331909_134945120)x3 |  | 14 |  | 25 | 60 | 27-yo female. Severe ID and primary psychosis, hypotonia, short stature and skeletal abnormalities, facial dysmorphism, cardiac murmur, strabismus, mild hearing loss, abnormalities of the posterior fossa, atrophy of corpus callosum, progressive myoclonus epilepsy |
| 19 | arr(7)x3  Trisomy 7 |  |  | 159 | 5 | 10 | 17mo-female.  Unusual,mosaic hyper/hypopigmented skin macules |
| 20 | arr(1)x3  Trisomy 1 |  |  | 249 | 5 | Not done | 1mo- female Complex cardiac abnormaility, tracheoesophageal fistula and oesophageal atresia, 13 paired ribs, VACTERAL spectrum |
