# Supplementary material for: Comparing saliva and blood for the detection of mosaic genomic abnormalities that cause syndromic intellectual disability
Source: Eur J Hum Genet. 2022 Nov 29;31(5):521–5. doi: 10.1038/s41431-022-01232-5 (PMC10172398; doi:10.1038/s41431-022-01232-5)
Supplement: Supplementary file 2 — Supplementary Table 2 [file 41431_2022_1232_MOESM2_ESM.docx]

**Supplementary Table 2:** Non mosaic genomic abnormalities detected in both blood and saliva sampling

| **MA Abnormality in blood and saliva [hg19]** | **Size of Genomic Imbalance (MB)** | **Terminal/trisomy/Interstitial** | **Recurrent/**  **Novel** | **Classification/Clinical significance** |
| --- | --- | --- | --- | --- |
| 1p36.23(8630602_8736745)x1 pat | 0.1 | Interstitial | N | VUS |
| 1p32.3(54,967,890-55,339,086)x3 | 0.4 | Interstitial | N | VUS |
| 1p21.2p21.1(101,551,926-102,270,082)x1 | 0.7 | Interstitial | N | VUS |
| 1q21.1q21.2( 146,501,348-147,828,939)x1 | 1.3 | Interstitial | R | Pathogenic-reduced penetrance |
| 1q24.2q25.1(169,869,344-173,883,864)x3 | 4.0 | Interstitial | N | VUS |
| 2p25.3(1,612,220-1,859,666)x3mat | 0.25 | Interstitial/partial gene | N | VUS |
| 2p16.3(51,171,962-51,280,121)x1 | 0.1 | Interstitial | N | Pathogenic |
| 2p16.3(50,863,273-50,972,117)x1 mat | 0.1 | Interstitial/intragenic | N | VUS |
| 2q13(111,392,259-113,100,014)x1 | 1.7 | Interstitial | R | VUS |
| 2q32.1(184786881_186173063)x1 | 1.4 | Interstitial | N | Likely Benign |
| 3p26.3(285,806-1,661,120)x3, 3q28q29(192,028,670-192,453,666)x1 | 0.4/1.4 | Interstitial | N | VUS |
| 3p21.1p14.3(54,285,016-57,908,707)x3dn | 3.6 | Interstitial | N | Pathogenic |
| 3p14.1(65878632_66519814)x3 pat | 0.6 | interstitial | N | VUS |
| 3q29(197401934_197871052)x1 dn | 0.5 | Terminal | N | Pathogenic |
| 4q21.21q21.22(81,397,872-82,843,970)x1dn | 1.4 | Interstitial | R | Pathogenic |
| 4q22.3(95,876,278-96,189,813)x1 | 0.3 | Interstitial | N | VUS |
| 4q34.1q35.1(176,073,580-186,940,878)x1,4q35.1q35.2(186,943,462-190,880,409)x3 | 11/4 | Terminal | N | Pathogenic |
| 5q11.1q11.2(49,562,999-52,574,651)x3 | 3.1 | Interstitial | N | VUS |
| 5q15(92,680,028-93,054,861)x1 | 0.4 | Interstitial | N | Pathogenic |
| 5q23.2(126,106,213-126,166,191)x3 pat | 0.06 | Interstitial | N | VUS |
| 5q33.1(150,743,405-151,612,227)x3 | 0.9 | Interstitial | N | VUS |
| 7q31.1(111,852,682-112,633,232)x3 | 0.8 | Interstitial | N | VUS |
| 8q24.3(145637148_145931897)x3 | 0.3 | Interstitial | N | VUS |
| 9q33.1(119,309,609-119,699,452)x1 | 0.4 | Interstitial | N | VUS |
| 9q34.3(138,662,465-138,785,699)x3pat | 0.1 | Interstitial | N | VUS |
| 10q25.1(108,469,093-109,599,439)x3 mat | 1.1 | Interstitial | N | VUS |
| 12q24.21(116,507,500-116,561,165)x1 dn | 0.05 | Interstitial | N | Pathogenic |
| 15q11.2q13.1(23,683,783-28,530,182)x1 | 4.9 | Interstitial | R | Pathogenic |
| 15q11.2(22,770,994-23,226,254)x1 | 0.5 | Interstitial | R | Pathogenic  -low penetrance |
| 15q11.2q13.1(23616115_28530182)x1 | 5.0 | Interstitial | R | Pathogenic-PWS |
| 16q24.3(89,341,500-89,386,808)x1 mat | 0.045 | Interstitial/partial gene | N | Likely Pathogenic |
| 17p12(14,098,277-15,457,054)x3 | 1.4 | Interstitial | R | Pathogenic |
| 17q25.1(71,836,274-72,667,520)x3pat | 0.8 | Interstitial | N | VUS |
| (18)x3 | 78 | Trisomy | R | Pathogenic |
| 19q13.2(39730501_40106388)x3 | 0.4 | Interstitial | N | VUS |
| 20p12.3(8,110,530-8,575,333)x3pat | 0.5 | Interstitial | N | VUS |
| 22q11.22(22,314,463-22,573,637)x0 | 0.3 | Interstitial | R | VUS |
| 22q13.33(51,128,648-51,214,796)x1 | 0.08 | Terminal/Partial gene | N | Pathogenic |
| 22q13.2q13.33(42,218,856-51,181,759)x1 | 9.0 | Terminal | R | Pathogenic |
| 22q13.31q13.33(46,550,106-51,169,045)x1 dn | 4.6 | terminal | Y | Pathogenic |
| Xp22.33(599,506-722,962)x3 | 0.1 | Interstitial | N | Likely Pathogenic |
| Xp22.31(6,456,940-8,126,718)x1 | 1.7 | Interstitial | R | Pathogenic |
| Xq28(148,702,179-149,313,435)x3 | 0.6 | Interstitial | N | VUS |
| Yp11.31q11.221(2,655,180-18,381,735)x2, Yq11.221q12(18,394,634-59,337,713)x0 | 18/41 | Terminal | N | Pathogenic |
