# Supplementary material for: Comparing saliva and blood for the detection of mosaic genomic abnormalities that cause syndromic intellectual disability
Source: Eur J Hum Genet. 2022 Nov 29;31(5):521–5. doi: 10.1038/s41431-022-01232-5 (PMC10172398; doi:10.1038/s41431-022-01232-5)
Supplement: Supplementary file 3 — Supplementary Table 3 [file 41431_2022_1232_MOESM3_ESM.docx]

| **Supplementary Table 3: Mosaic genomic abnormalities detected in both blood and saliva** | | | | |
| --- | --- | --- | --- | --- |
| **MA Abnormality in blood and saliva [hg19]** | **Mosaicism% (blood/saliva)** | **Terminal/ Interstitial/ Partial chromosome/Whole chromosome/ESAC/Chimerism** | **Recurrent/Novel** | **Clinical classification** |
| (X)x1~2,(Y)x1 | (25/40) | Whole chromosome | R | Pathogenic |
| (14)x2 hmz | (15/15) | Whole chromosome Copy neutral | R | Pathogenic |
| (9p)x2 hmz | (10/10) | Terminal/Whole arm/ Copy neutral | R | VUS |
| 14q32.11q32.33(90782706_107274052)x1 | (30/30) | Terminal/Partial chromosome  (16MB del) | N | Pathogenic |
| 18q21.2q23(48986514_78077248)x1 | (5/43) | Terminal/Partial chromosome  (29MB del) | N | Pathogenic |
| 17p11.2q11.2(21295270_26516145)x3dn,17q11.2(26531353_26883313)x4dn,17q11.2(26909156_27924179)x3dn | (85/85) | Mosaic ESAC  (6.6 MB gain) | R | Pathogenic |
| chi(1-22,X)x2,(1-22)x2,(XY)x1 | (70/30) | Chimerism | R | Pathogenic |

ESAC=extra structurally abnormal chromosome
